# Effects of mosquito-proofing storm drains on adult and larvae mosquito abundance: Protocol of the IDAlErt storm drAin randomiSed controlled trial (IDEAS)

**DOI:** 10.1016/j.mex.2024.103102

**Published:** 2024-12-11

**Authors:** Marina Treskova, Tomás Montalvo, Joacim Rocklöv, Charles Hatfield, Frederic Bartumeus, Shouro Dasgupta, João Encarnação, Rachel Lowe, Jan C. Semenza, Pascale Stiles, Jordi Noya, Andrea Valsecchi, Till Bärnighausen, John R.B. Palmer, Aditi Bunker

**Affiliations:** a Heidelberg Institute of Global Health (HIGH), Heidelberg University Hospital, Heidelberg University, Heidelberg, Germany; b Interdisciplinary Center for Scientific Computing (IWR), Heidelberg University, Heidelberg, Germany; c Department of Public Health and Clinical Medicine, Section of Sustainable Health, Umea University, Umeå, Sweden; d Agència de Salut Pública de Barcelona, Barcelona, Spain; e CIBER Epidemiología y Salud Pública (CIBERESP), Calle Monforte de Lemos 5, 28029 Madrid, Spain; f Theoretical and Computational Ecology Group, Centre d'Estudis Avançats de Blanes (CEAB-CSIC), Girona, Spain; g Institució Catalana de Recerca i Estudis Avançats (ICREA), Barcelona, Spain; h CREAF Cerdanyola del Vallès, Spain; i Centro Euro-Mediterraneosui Cambiamenti Climatici (CMCC), Venice, Italy; j Graham Research Institute on Climate Change and the Environment, London School of Economics and Political Science (LSE), London, United Kingdom; k Irideon, Barcelona, Spain; l Barcelona Supercomputing Center (BSC), Barcelona, Spain; m Centre on Climate Change & Planetary Health and Centre for Mathematical Modelling of Infectious Diseases, London School of Hygiene & Tropical Medicine (LSHTM), London, United Kingdom; n Department of Political and Social Sciences, Universitat Pompeu Fabra, Barcelona, Spain; o Institut d'Investigació Biomèdica Sant Pau (IIB SANT PAU), Sant Quintí 77-79, 08041 Barcelona, Spain; p Heidelberg Institute for Geoinformation Technology gGmbH (HeiGIT), Heidelberg University, Heidelberg, Germany

**Keywords:** Adaptation, *Aedes*, Climate change, *Culex*, Mosquito intervention, Urban infrastructure, Vector-borne

## Abstract

*Aedes* and *Culex* mosquitoes, known for spreading arboviruses like dengue and West Nile, thrive in cities, posing health risks to urban populations. Climate change can create suitable climatic conditions for these vectors to spread further in Europe. Cities contain numerous landscape and infrastructure elements, such as storm drains, that allow stagnant water build-up facilitating mosquito breeding. Modifying urban infrastructure to prevent water accumulation can reduce mosquito populations, but evidence is limited. The Public Health Agency of Barcelona, Spain, introduced a structural modification of storm drains to prevent water accumulation. Together with the Agency, we designed a randomised controlled trial (RCT) to experimentally assess the effectiveness of these modifications on adult *Aedes albopictus* and *Culex pipiens* populations. It is a parallel-arm RCT with equal randomization of 44 drains to receive mosquito-proofing modifications (intervention) or not (control). Primary outcomes are adult mosquito counts and secondary outcomes are larvae and mosquito presence, assessed weekly at each drain until no mosquitoes are detected. Data analyses include generalised linear mixed models to estimate the time-averaged and highest intervention effects, subgroup and sensitivity analyses. The trial results will guide a city-wide expansion of the storm drain modifications and provide valuable evidence to enhance existing vector control measures.

## Graphical abstract

**Figure:**
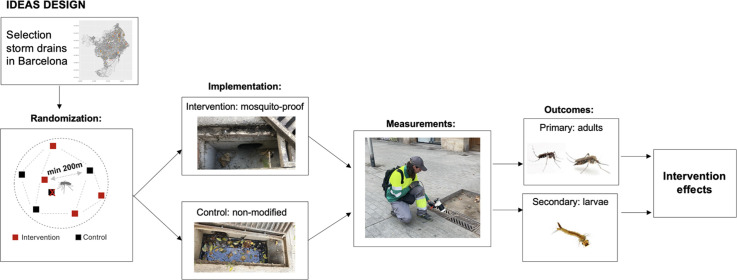

Specifications table

**Table:** 

Subject area:	Environmental Science
More specific subject area:	Public health, mosquito control, urban design, urban climate change adaptation
Name of your protocol:	The effectiveness of mosquito-proof storm drains on adult mosquito and mosquito larvae abundance: protocol of the IDAlErt storm drAin randomiSed controlled trial (IDEAS) in Barcelona, Spain
Reagents/tools:	Entomological aspirator: Improved Prokopack Aspirator Model 1419 from John W. Hock Co., powered by a 12 v 9.0 Ah valve-regulated, rechargeable sealed lead battery.
Experimental design:	This is a protocol for a randomised controlled trial (RCT) to investigate the effects of mosquito-proofing storm drains in Barcelona on the counts of adult *Aedes albopictus* and *Culex pipiens* mosquitoes. Using a parallel-arm design, we randomise 44 drains to receive mosquito-proofing modification (intervention) or not (control). The primary outcome is the combined counts of total adult *Ae. albopictus* and *Cx. pipiens* mosquitoes captured using an entomological aspirator during the trial period. Secondary outcomes include the presence of either mosquito larvae or adult mosquitoes (binary). The measurement of primary and secondary outcome takes place weekly until no mosquitos are detected. The effects of the intervention are estimated comparing mosquito-proofed and control drains using generalised linear mixed models.
Trial registration:	This trial is registered as a trial in Open Science Framework Registry. Registration DOI https://doi.org/10.17605/OSF.IO/JZQAH
Ethics:	The IDEAS trial does not involve human subjects and does not pose any significant health risks to the community. We leveraged the existing program for storm drain modifications by the ASPB and assigned the intervention by randomly selecting drains from a pool of storm drains eligible for modification. To date, no cases of locally transmitted mosquito-borne diseases affecting humans have been reported in the city of Barcelona, but the ASPB applies existing surveillance protocols for mosquito-borne arboviruses, including the necessary anti-larval and/or adulticide treatments if a human case is detected. Any such instances that affect drains included in the trial will be accounted for in the analysis. The trial does not include experiments with animals.
Value of the Protocol:	1. This protocol describes in detail the aims and methods of a randomized control trial designed together with a governmental public health agency to evaluate the effects of an urban infrastructure intervention on *Aedes albopictus* and *Culex pipiens* mosquito counts. 2. This study exemplifies a transdisciplinary collaboration between governmental stakeholders and universities to co-produce robust evidence to inform decision-making regarding the upscale of the intervention. 3. This trial will investigate an urban infrastructural intervention that potentially has far reaching implications in safe-guarding public health against mosquito-borne diseases and can be considered as an upstream prevention measure.

## Background

Cities are becoming increasingly vulnerable to vector-borne diseases (VBD) due to climate change [1,2]. Mosquitoes belonging to the *Aedes* and *Culex* genera, known carriers of arboviruses such as yellow fever, Zika, chikungunya, West Nile, and dengue viruses, are sensitive to climate, including temperature, humidity, and precipitation [3]. *Aedes* and *Culex* species are well adapted to human settlements, utilising stagnant water bodies for egg-laying and larval development [[4], [5], [6]]. Urban sewage systems with stagnant water or poor drainage propagate the breeding and proliferation of *Aedes* and *Culex* mosquitoes [7,8], creating opportunities for mosquitoes to lay eggs and complete their life cycle [8].

Mosquito vectors, including the *Culex pipiens* [4] and *Aedes albopictus* [9,10] species, present health risks throughout Europe. *Cx. pipiens* is native to the continent; *Ae. albopictus* is currently established in Europe's Mediterranean countries, Switzerland, Germany, Hungary, Romania, Moldova, Bulgaria, Serbia, Kosovo, Macedonia, Ukraine, Russia, Georgia, and Armenia [11]. In Spain, *Ae. albopictus* was first detected in 2004 near Barcelona [12] and has spread into eastern and northern provinces and farther inland [[13], [14], [15],^11^]. Despite active monitoring and control efforts, mosquito populations continue to proliferate. Changes in climate coupled with the availability of new ecological niches and immunologically naïve populations [1,16] pose challenges in controlling vector and pathogen spread. Adaptive strategies, including modifying urban infrastructure, can co-beneficially act as upstream preventative interventions against VBDs by affecting vector populations. Infrastructural modification can be costly, and, therefore, require causal evidence of their effectiveness before implementation and scaling.

Barcelona is a Mediterranean city with 1.7 million inhabitants, in which *Ae. albopictus* and *Cx. pipiens* populations are primarily managed through applying biocides in breeding sites [17,6]. In 2018, a dengue outbreak was detected in Barcelona, originating from the arrival of a viremic passenger into an area of *Ae. albopictus* activity [18]. This revealed that ongoing surveillance and control measures were insufficient for long-term reduction in breeding sites. Recognising the need for a paradigm shift, the Barcelona Public Health Agency (ASPB) began piloting an infrastructural modification to storm drains–‘mosquito-proofing’–that diverts stormwater into sewers rather than filtering it through the sand. Mosquito-proofing storm drains in urban public areas can: (i) minimise the establishment of breeding sites to future-proof the arrival of vectors [17], (ii) sever mosquito proliferation at existing breeding sites, and (iii) reduce the need for traditional biocide treatment in high-mosquito-activity drains [19].

Preliminary observations of mosquito-proofing storm drains in Barcelona show reduced mosquito larvae activity, reduced drain flooding and decreased reliance on biocide treatment [17]. Although initial results are promising, we require evidence from robust causal impact evaluations for widespread implementation of mosquito-proof storm drains. We are therefore conducting a randomised controlled trial (RCT) to investigate the effect of mosquito-proofing storm drain modifications on *Ae. albopictus* and *Cx. pipiens* abundance in Barcelona. RCTs provide a gold standard for causal inference evaluation and allow the observed effects to be attributable to the intervention [20]. This RCT will overcome the limits of the previous observational study, provide robust evidence of the effects of the storm drain modifications on the mosquito numbers in Barcelona, and support decision-making by the city government regarding a widescale implementation of the modification.

## Description of protocol

### Objectives

This protocol describes the aims and the methods of the IDAlErt storm drAin randomiSed controlled trial (IDEAS) in Barcelona, Spain. In the IDEAS trial, we aim to investigate the effect of urban mosquito-proofing storm drain modifications on *Ae. albopictus* and *Cx. pipiens* numbers found in these breeding sites. We hypothesise that modified storm drains will exhibit lower adult and larval mosquito abundance than unmodified drains. Whereas the observational study by Montalvo et al. [17] shows that drain modification reduces water accumulation and mosquito larval activity within the modified drains, it could be confounded by uncontrolled factors. Our study, thus, aims to ascertain the effectiveness of this infrastructure intervention within an experimental study that addresses the confounding factors. Our findings will add evidence to guide recommendations for implementing this intervention and strengthen vector control efforts.

This study is part of the larger Horizon Europe project “Infectious Disease decision-support tools and Alert systems (IDAlert)” (https://idalertproject.eu/). The IDAlert project aims to develop long-lasting solutions to reduce climate change-induced infectious disease risks through collaborations between multiple research institutes and decision-making stakeholders [21]. This trial involves direct collaboration between the Barcelona Public Health Agency (Agència de Salut Pública de Barcelona, ASPB), Universitat Pompeu Fabra (UPF), and Heidelberg University (UKHD). Ultimately, the evidence generated from this trial will inform decision-making regarding expanding storm drain modifications as a sustainable tool for VBD prevention.

### Trial design

**Fig. 1:**
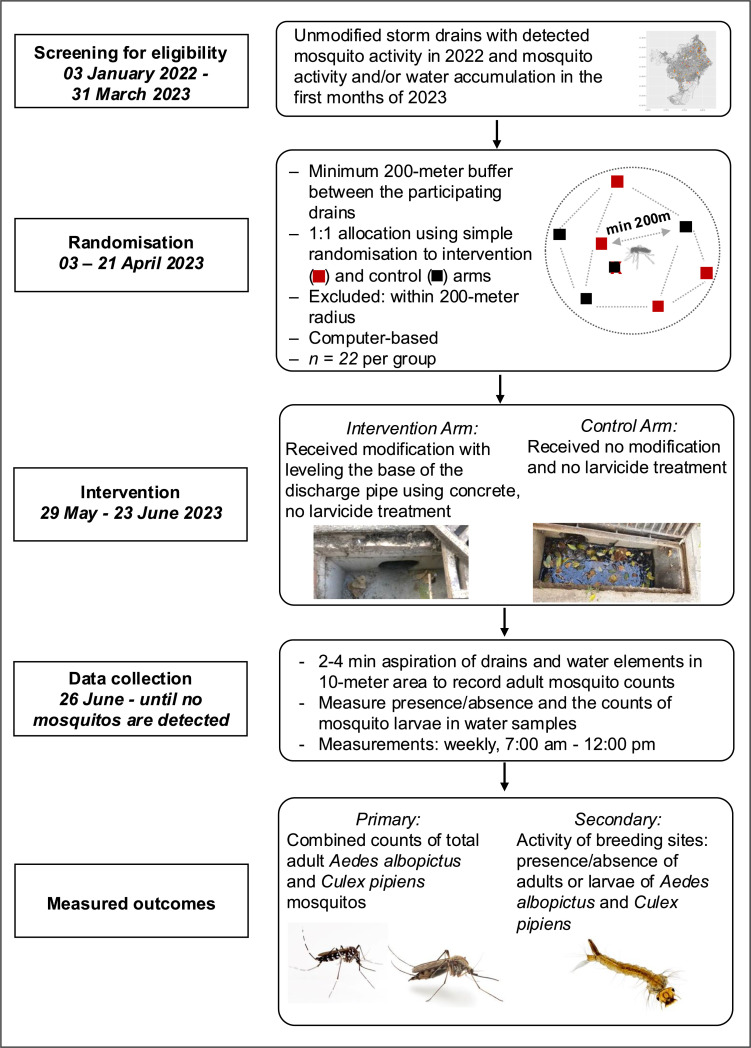
The flow chart of the IDAlErt storm drAin randomiSed controlled trial (IDEAS) in Barcelona, Spain.

IDEAS trial is a causal evaluation of a structural modification of urban storm drains envisioned by the Barcelona city administration to address mosquito-related threats and nuisances. We leverage the planned implementation to conduct a robust experimental study. Together with the governmental stakeholders, we designed a trial outlined in this protocol. We conduct a parallel-arm randomised controlled trial where eligible storm drains are randomised 1:1 to receive the mosquito-proofing intervention (intervention arm) or not (control arm). We selected eligible drains based on the detection of mosquito activity in 2022/2023 by the Barcelona Public Health Agency. We created a 200-meter zone around each eligible drain to account for mosquito dispersal capacity [[22], [23], [24], [25]] and minimise the risk of contamination across trial arms. We will measure outcomes in the intervention and control storm drains and in water bodies in the vicinity of the drains to capture mosquitoes that may be flying to experimental drains from nearby breeding sites. The overall design is illustrated in Fig. 1.

### Study setting

We are conducting the IDEAS trial from 26 June 2023 until no mosquitoes are detected in 2023, capturing the mosquito season in Barcelona, Spain. The intervention is implemented by ASPB and the Barcelona Cicle de l'Aigua (BCASA)–the city's water infrastructure authority. All storm drains included in this trial are located in the city's public spaces. The trial does not involve private property.

### Eligibility criteria

**Fig. 2:**
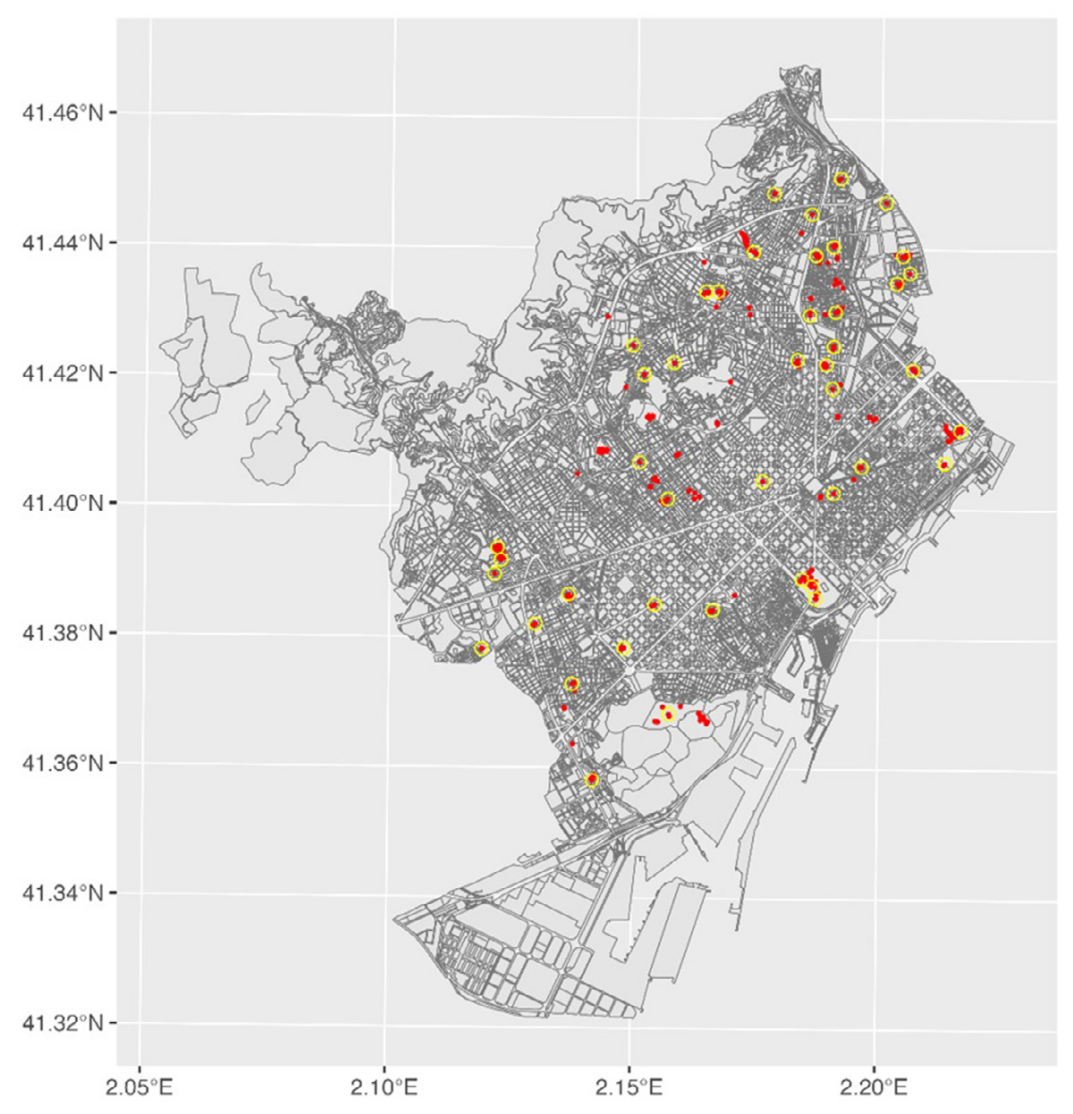
The red dots depict the location of storm drains eligible for sampling based on activity. The yellow rings encompassing the red dots represent the drains (*n* = 44) we selected for IDEAS. The source of the base map in grey is from “Ground uses of the city of Barcelona”, CC BY 4.0, Ajuntament de Barcelona, 2016, available at https://opendata-ajuntament.barcelona.cat/data/en/dataset/mapa-usos-sol-wms).

We defined two eligibility criteria for including storm drains in the IDEAS trial based on ASPB's mosquito surveillance and control program (Fig. 2). We first selected storm drains in which mosquito activity was detected during 2022. The sample of participating drains was augmented by selecting drains in which potential activity–defined as the accumulation of standing water–was detected from January to March 2023. We also required that selected drains must be at least 200 m from one another. Selection details are given in S1 File (Section: Allocation of intervention and concealment mechanism).

### Interventions

**Fig. 3:**
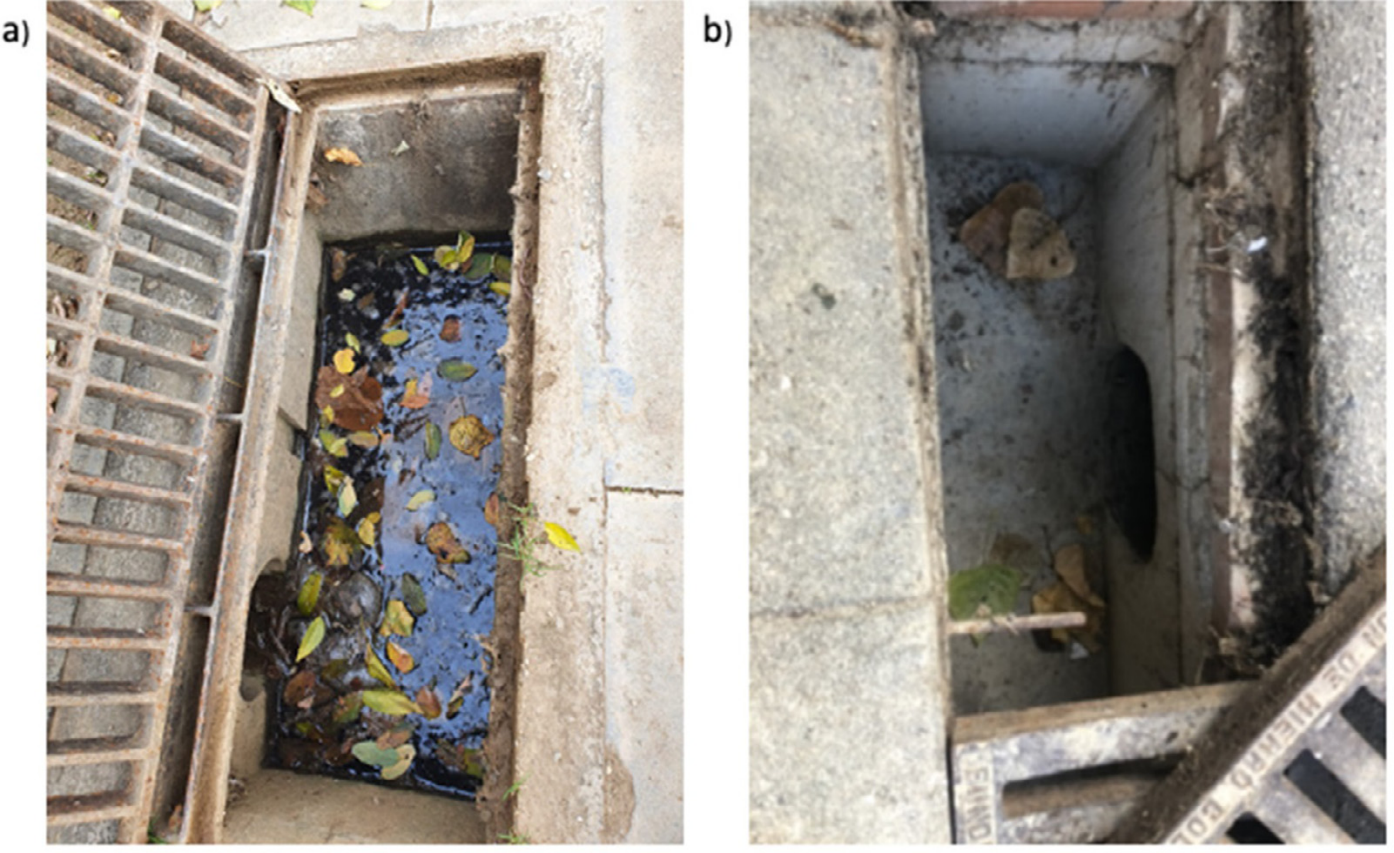
Panel a) depicts an unmodified storm drain with stagnant water, and panel b) depicts a modified storm drain. Source: We obtained photos from [17] with permission for re-use.

Our intervention, i.e., mosquito-proofing modification of storm drains, is implemented by our partners at the Barcelona water infrastructure authority, BCASA. We first place honeycomb bricks at the base of the drain to provide structural integrity and subsequently added concrete to create a gradient. We connect the lowest point of the gradient to the discharge pipe, enabling the water to flow down the gradient to prevent water accumulation (Fig. 3). This rapid and low-cost mosquito-proofing intervention is intended to eliminate stagnant water in the catchment drain, rendering the drain unsuitable for mosquito reproduction.

We did not modify the 22 storm drains in the control arm, which served as mosquito breeding sites. We do not apply larvicides to either the treatment or control drains during this trial unless there is a confirmed arbovirus case detected in proximity to the drain(s). In such circumstances, ASPB will activate standard treatment protocols for protecting public health [26]. ASPB is following existing treatment protocols for all non-participating drains.

### Criteria for excluding or modifying allocated interventions

Upon inspection, our partners at ASPB and the water infrastructure authority excluded drains that did not fit the inclusion criteria. We applied three exclusion criteria for selecting storm drains in our trial: (i) previously modified drains that were unreported in the public record, (ii) inaccessible drains in construction areas or where obstructed by public infrastructure, and (iii) obstruction from cars parked on top of the drains.

### Outcomes

Our objective in this trial is to assess the impact of storm drain modifications on *Ae. albopictus* and *Cx. pipiens* mosquito numbers inside intervention and control drains. Our primary outcome is the combined total counts of adult *Ae. albopictus* and Cx. *pipiens* mosquitoes sampled at each drain. The secondary outcome is drain activity defined as the presence of either adult mosquitoes or *Ae. albopictus* and *Cx. pipiens* larvae (L1-L2, L3-L4, and pupae stages) in drain water inside the drains. We sample water elements to collect adult mosquitoes and larvae at these alternative breeding sites. We also measured the depth of water (cm) in corresponding drains. One trained ASPB technician measures the primary and secondary outcomes starting on 26 June 2023 and continuing until no mosquitos are detected, visiting each participating drain once per week.

### Power calculation

We based our power calculation on the primary outcome to detect at least a 35% difference in mosquito count between the intervention and control arm at a significance level (p-value) of 5%. We explored within-class correlation and the statistical distribution of our primary outcome using data on adult mosquitoes collected in 2022 by ASPB using data from artificial intelligence (AI)-driven smart traps [27]. Our analysis showed independence between weekly measurements, and the data followed a Negative Binomial distribution. Therefore, we performed the power calculation in *R* [28] using the *power_NegativeBinomial* function of the PASSED package ([28,29]). Assuming a minimum detectable difference in mosquito counts of 35% between the intervention and control arms and a significance level of 5%, we estimate a statistical power of at least 90% value for our sample of 44 drains. Further details of the power calculation are given in S1 File (Section: power calculation).

### Assignment of the intervention

#### Sequence generation

From the pool of 158 eligible drains, we allocated 44 drains 1:1 to the intervention or control arm, respectively, using a simple randomisation algorithm we developed in *R* [28]. The remaining drains within 200 m of a trial drain were eliminated from the eligibility pool. The process was repeated, alternating assignments between intervention and control arms until no drains remained for selection. We present details in S1 file (Section: Allocation of intervention and concealment mechanism).

#### Implementation

We wrote an *R* script for the selection of drains. The intervention allocation arm (i.e., intervention or control arm) was randomly assigned the letter code A or B, and two sets of shapefiles for the locations of selected drains were generated. The first showed both the intervention arm and letter code assignment for ASPB and BCASA, which are responsible for implementing the storm drain modification and collecting entomological data; the second included only the letter code which we shared with the blinded biostatisticians conducting the analysis. ASPB and BCASA used the allocation results and inspected the drains assigned to the intervention arm for the feasibility of the modification. If the modification was not possible, we assigned the next eligible drain.

## Data collection

### Assessment and collection of outcomes

**Fig. 4:**
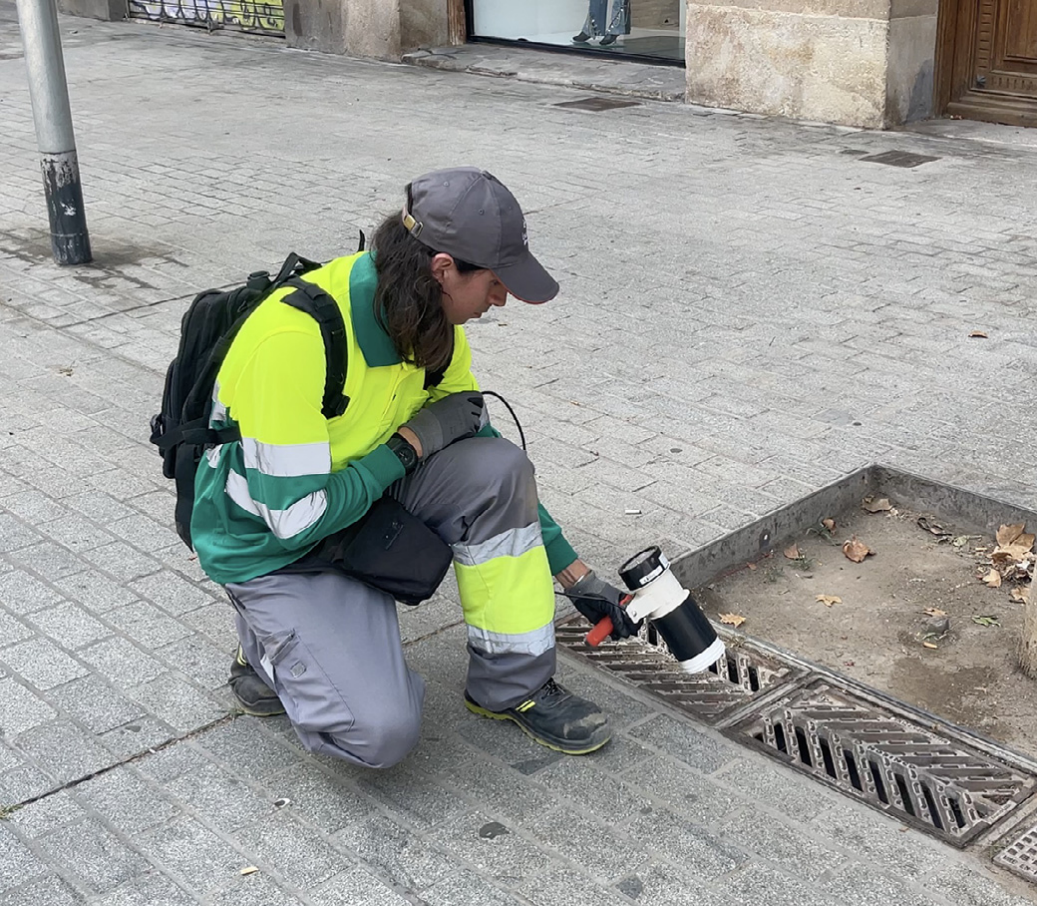
Photo of the ASPB field technician with an entomological aspirator. Photo by Chloe Chavardes.

We have scheduled an ASPB field technician to visit participating drains weekly between 7am and 12pm starting on 26 June 2023 and continuing until no mosquitos are detected. During each visit, our technician will collect data on the primary and secondary outcomes and the presence and depth of water in the storm drains. We have established a sampling protocol that our technician will follow, which includes the use of protective gear, including gloves and sturdy shoes. The equipment for the sample collection will remain distal from the drainage element (Fig. 4).

### Adult mosquito collection

Our ASPB field technician uses an entomological aspirator (Improved Prokopack Aspirator Model 1419 from John W. Hock Co., powered by a 12 v 9.0 Ah valve-regulated, rechargeable sealed lead battery) to collect adult mosquitoes at specified drains. Wearing gloves, the technician assembles the aspirator consisting of the vacuum and the sample collection vesicle on-site. Holding the aspirator at a minimum one-meter horizontal distance from the drain, the technician approaches the drain and prepares for mosquito capture. The technician stomps on the drain to disturb resting mosquitoes and prompts them to take flight while simultaneously using the aspirator to capture escaping mosquitoes or those resting nearby. The technician subsequently opens the storm drain and systematically vacuums the interior space and walls to capture any resting mosquitoes. If water is present in the drain, the technician vacuums the walls closest to the water at arm's length, as mosquitoes tend to rest in these areas. If there is no water, the technician vacuums at arm's length to cover the entire interior space.

### Larval sampling

Our technician collects a water sample from the drain to assess the presence of mosquito larvae. After submerging the dipping tool (volume 0.5 L with a 1.5-meter long handle) vertically into the water, our technician withdraws the dipping tool to pour the water sample into a designated container [17]. Using pipettes, our technician individually extracts larvae and transfers them into vials. When the water level is low and predominantly contains organic matter such as dirt and debris, we use a hand collector to gather the larvae. We send the vials to the laboratory for species determination. If possible, our technician approximates the number of larvae in the drain stratified by larvae development stages (i) L1-L2 stages, and (ii) L3-L4 stages, and (iii) pupae.

### Laboratory evaluation and storage of biological specimens

Following collection, we transfer the samples to the ASPB laboratory, where they will be stored at −20 °C. We morphologically identify samples using a stereoscope to classify them by species (in the case of *Culex*, to species complex) and sex. We store mosquito samples (by assigning a unique identifying number) for at least two years following the completion of the trial.

### Buffer zones

We investigate the effects of modifying main trial storm drains on adult mosquito counts in alternative mosquito breeding sites. These include water infrastructure features within a 10-meter radius around the main trial drains.

## Statistical methods

Our main analyses are complemented by sensitivity analyses to establish the causal effects of mosquito-proofing storm drains on primary and secondary outcomes. The main analyses focus on the time-averaged and highest effects of the intervention on primary and secondary outcomes. For the combined total adult *Ae. albopictus* and *Cx. pipiens* mosquito counts, i.e., our primary outcome, we assume a Negative Binomial distribution and within-subject independence based on our analyses for the sample size and power calculation. We quantify the effects of the intervention vs. control drains on the primary outcome using a generalised linear mixed model (GLMM), applying random effects for storm drains. We aim to report one effect estimate that is time-averaged and one for September 2023 to demonstrate the highest estimated effect of the intervention. We selected September based on the 2022 data showing the peak of mosquito numbers in Barcelona (see S1 File, the sample size and power calculation section). We further investigate intervention effects by stratifying total adult mosquito counts by species (*Ae. albopictus* and *Cx. pipiens*), sex and month. We report statistically significant effect estimates when the *p*-value is below 0.05.

In our sensitivity analyses, we assess the distribution of land cover characteristics between the control and intervention drains. We also evaluate the assumptions underlying regression analyses, using historical data. Further, we account for variations in weather elements such as relative humidity and temperature, which are assumed to be spatially similar across both arms but could vary based on the timing of measurements. These adjustments aim to reduce uncertainty in the intervention effect estimate. If land cover characteristics are unevenly distributed between the arms, we refine the models to account for land cover effects. To explore our assumptions, we first re-evaluate within-subject correlation, fitting the generalised estimating equations model (GEE) to our data. Second, we explore the primary outcome distribution, fitting intercept-only generalised linear models with Poisson and zero-inflated Poisson distributions. In case of deviation from the assumed distribution and/or independence, we conduct further sensitivity analyses using appropriate modelling approaches.

Prior to conducting regression analyses, we clean the data, tabulate descriptive statistics, and perform data visualisation [30].

All data analyses will be performed using *R* [28]. Scripts for data analyses will be checked and validated by the two analysts and posted in an open GitHub repository using R-markdown [31] following publication of trial results.

## Summary

Urban public health and environmental authorities face pressure to address emerging infectious disease threats in the face of climate change to protect inhabitants. Interventions to combat these threats should be based on robust scientific evidence. IDEAS utilises gold-standard RCT methodology to test the effects of mosquito-proofing storm drain modifications on *Ae. albopictus* and *Cx. pipiens* mosquito populations in the storm drains. These vectors are of particular importance for public health because climate change is shifting and expanding their geographical range, altering the life cycle, vectorial capacity and transmission processes, increasing the risk of mosquito-borne disease in urban populations [19]. Cities in which *Ae. albopictus* and *Cx. pipiens* mosquitoes are present require that mosquito surveillance and control methods be ramped up, and many cities where they are not present should prepare for their arrival.

Storm drains in public areas are typical sites for stagnant water to accumulate, propagating mosquito larvae development. In recognising this, we are modifying urban drain infrastructure to curtail the upstream proliferation of mosquitoes, which can potentially yield substantial benefits. For example, we can reduce the long-term operational costs associated with surveillance and control initiatives, including the use of biocides to treat drains. Mosquito-proofing storm drains is likely to optimally function when complimented with sanitation practices, efficient sewage management, and comprehensive mosquito control measures. These remain indispensable components in the ongoing effort to mitigate the breeding and spread of mosquitoes in urban environments.

The measurement duration, methods, and timing of outcome assessment we selected were intended to maximise statistical power, considering the constraints of a densely populated urban environment. The decision to aspirate adult mosquitoes and use larval dippers to measure mosquito larvae was supported by entomologists at ASBP, who are the most experienced with Barcelona storm drains. The aspirators we have been using in the field are designed to collect resting mosquitoes in confinement. We did not place mosquito traps at the drains because of their impractical size (around 0.5 m high) and potential to be displaced by pedestrians or stolen in public areas. Aspiration allows for consistent capture of resting mosquitoes at the time of measurement and the determination of respective mosquito species at the drain and elements in close proximity. Although we will not determine the water body in which larval growth occurred, collecting information on focal drains and their 10-meter surroundings can potentially help us understand how mosquitoes shift to nearby breeding sites when one site is eliminated. Future studies can be designed to investigate the effects of storm drain modification vs. no storm drain modification of all drains in larger urban quadrants. While our IDEAS trial is not intended to evaluate the effects of a large-scale drain modification, it is a necessary preliminary step for the city administration to consider broader implementation.

Governmental stakeholders such as ASPB and Barcelona's water infrastructure authority (BCASA) contributed significantly to trial design and execution. These agencies have established protocols for informing and engaging citizens affected by public infrastructure projects. These were adhered to in our IDEAS trial. We will distribute the IDEAS trial findings with stakeholders, including community members, to inform the public on outcomes related to storm drain modification.

## Protocol validation

*Not applicable*.

## Limitations

IDEAS has potential limitations stemming from the nature of the intervention. We likely demonstrate the effectiveness of the intervention that is context-dependent. Barcelona is a historic city with a unique urban design. The infrastructural intervention that works in the Barcelona setting may not necessarily be applicable or effective in another European city. IDEAS will demonstrate the effects of storm drain modifications on mosquito abundance in Barcelona. The generalizability of our findings to other urban contexts may be limited owing to differences in population densities, infrastructural layouts (including drain infrastructure), transportation systems, waste management practices, street cleaning routines, climate, and baseline mosquito population levels in public spaces in other regions. Future multisite trials are required to test the effectiveness of storm drain modifications in contexts outside of Barcelona to address generalizability.

Further, although the AI-driven mosquito traps are ideal for capturing mosquito abundance longitudinally, it's not feasible to leave the traps at the drains for extended periods of time. We mitigated this limitation by calculating our sample size to account for the cross-sectional outcome collection using the entomological aspirators. Our trial steering committee will likely recommend ending our trial once statistically significant effects (positive or negative) have been detected. Following IDEAS, ASPB will continue long-term monitoring of intervention and control drains through routine surveillance. We will, therefore, continue assessing whether the storm drain modification remains effective over time and acquire insights about potential maintenance requirements.

Despite these limitations, IDEAS is an interdisciplinary project involving public health (infectious disease epidemiology and trial design), urban design (mosquito-proofing storm drain implementation), and public policy (government stakeholders as recipients of evidence) domains. We have capitalised on a unique opportunity to collaborate with the Public Health Agency (ASPB) in Barcelona to randomise drains to receive a mosquito-proofing intervention (or not). Using the RCT methodology, our goal is to conduct a causal impact evaluation to provide evidence for policymakers on the effectiveness of this mosquito-proofing infrastructure modification on mosquito abundance in Barcelona, Spain, providing a model for future intervention projects in the European Union and beyond.

## CRediT author statement

AB, JP, TM, JS, RL, JR, MT, FB, and TB contributed significantly to the trial's conceptualisation. AB is the principal investigator of the trial. MT, JP, AB, TB and JR, contributed to the statistical design and power calculations. JE contributed the smart trap data used for the power analysis and study design. MT, JP and AB led the writing of the manuscript. CH, PS, RL, and SD contributed to historical data analysis and drafting of the protocol. TM, AV, FB, and JP conceptualised and outlined the procedures of the data collection. TM and AV provided training for the technician. All authors have read, provided input to, and approved the final version of the manuscript.

## Declaration of competing interest

The authors declare that they have no known competing financial interests or personal relationships that could have appeared to influence the work reported in this paper.

## Data Availability

Following publication of the IDEAS trial results, we will publicly release data on an open-access repository following the FAIR (Findable, Accessible, Interoperable, Reusable) guidelines.

## Appendix Supplementary materials

Image, application 1
