# Supplementary material for: Effects of mosquito-proofing storm drains on adult and larvae mosquito abundance: Protocol of the IDAlErt storm drAin randomiSed controlled trial (IDEAS)
Source: MethodsX. 2024 Dec 11;14:103102. doi: 10.1016/j.mex.2024.103102 (PMC11755014; doi:10.1016/j.mex.2024.103102)
Supplement: Supplementary file 1 [file mmc1.docx]

## Supplementary Material 1

## Effects of mosquito-proofing storm drains on adult and larvae mosquito abundance: protocol of the IDAlErt storm drAin randomiSed controlled trial (IDEAS)

## Marina Treskova^1,2,3^, Tomás Montalvo^4,5,15^, Joacim Rocklöv^1,2,3^, Charles Hatfield^1,16^, Frederic Bartumeus^6,7,8^, Shouro Dasgupta^9,10,^ João Encarnação^11^, Rachel Lowe^12,7^, Jan C. Semenza^1,2,3^, Pascale Stiles^1^, Jordi Noya^15^, Andrea Valsecchi^4^, Till Bärnighausen^1^, John Palmer^14^, Aditi Bunker^1^

Affiliations

1. Heidelberg Institute of Global Health (HIGH), Heidelberg University Hospital, Heidelberg University, Heidelberg, Germany
2. Interdisciplinary Center for Scientific Computing (IWR), Heidelberg University, Heidelberg, Germany
3. Department of Public Health and Clinical Medicine, Section of Sustainable Health, Umea University, Umeå, Sweden
4. Agència de Salut Pública de Barcelona, Barcelona, Spain
5. CIBER Epidemiología y Salud Pública (CIBERESP), Calle Monforte de Lemos 5, 28029 Madrid, Spain
6. Theoretical and Computational Ecology Group, Centre d'Estudis Avançats de Blanes (CEAB-CSIC), Girona, Spain
7. Institució Catalana de Recerca i Estudis Avançats (ICREA), Barcelona, Spain
8. CREAF Cerdanyola del Vallès, Spain
9. Centro Euro-Mediterraneo sui Cambiamenti Climatici (CMCC), Venice, Italy
10. Graham Research Institute on Climate Change and the Environment, London School of Economics and Political Science (LSE), London, United Kingdom
11. Irideon, Barcelona, Spain
12. Barcelona Supercomputing Center (BSC), Barcelona Spain
13. Centre on Climate Change & Planetary Health and Centre for Mathematical Modelling of Infectious Diseases, London School of Hygiene & Tropical Medicine (LSHTM), London, United Kingdom
14. Department of Political and Social Sciences, Universitat Pompeu Fabra, Barcelona, Spain
15. Institut d’Investigació Biomèdica Sant Pau (IIB SANT PAU), Sant Quintí 77-79, 08041 Barcelona, Spain
16. Heidelberg Institute for Geoinformation Technology gGmbH (HeiGIT), Heidelberg University, Heidelberg, Germany

Supplementary Material 1 describes details of the methods outlined in the protocol.

### Allocation of intervention and concealment mechanism

We selected the drains as follows. We started with 165 storm drains in which ASPB had detected mosquito activity during 2022 and randomly selected 42 drains from these using the sampling procedure described below. We then compiled a list of 158 drains in which activity and/or standing water had been detected during the first months of 2023, and that had not already been selected in the first round. Following the same sampling procedure we selected 30 additional drains from this new list, bringing the total to 72 selected drains. However, during the modification and the initial weeks of sampling, we determined that 24 of these could not be used due to the exclusion criteria described in the main text. Out of the remaining 48 drains, 22 had been assigned to the intervention arm and 26 to the control arm. We then selected 4 of the control drains at random and removed them from the study to give us a balanced set of 22 treatment and 22 control drains. We eliminated these rather than converting two of them to treatment drains because modifications would not have been possible at that stage. Thus, the final sample size of participating drains is 44 drains.

We have written the buffered_sample function for selecting drains, which works as follows:

1. From the set of eligible drains, choose one at random (simple randomisation), and assign it to the treatment or control arm.
2. Calculate the distances between the selected drain and the pool of drains and remove from the eligible set any drains that are 200 meters or less from the selected drain
3. Repeat steps 1-2, alternating assignments between treatment and control, until there are no more drains in the eligible set.
4. If the final sample contains an odd number of drains, eliminate the last one from the sample.

The code uses a randomly generated seed value for the randomisation, and it saves this value so that the samples can be subsequently replicated. The code and related files can be accessed at [**https://github.com/IDAlert/storm_drain_selection**](https://github.com/IDAlert/storm_drain_selection/releases/tag/v2.03)**.**

### Sample size and power calculation

This trial is powered in relation to the primary entomological outcome, i.e., combined total adult *Aedes* and *Culex* mosquito counts. Based on the prior test run of the sampling and allocation algorithms and drain inspections, we identified that at most 44 storm drains could be selected for the trial under the 200-m spacing constraint. Therefore, we performed power calculations for the available sample size as opposed to a sample size calculation.

We obtained the data for the power calculations from the 2022 records of mosquitoes entering five AI-driven "smart traps" located in Barcelona. These traps, designed and produced by Irideon SL (Barcelona, Spain), consist of an adult mosquito suction trap fitted with an opticoelectronic sensor that mosquitoes pass through when entering the trap (28). The sensor relays data near-real-time to a server that classifies the mosquitoes according to genus and sex. Although we expect that our outcome sampling method captures mosquitoes more quickly than the traps, we use the trap captures as a proxy here on the assumption that the capture distributions will be similar. Using the smart trap data, which includes the exact time that each mosquito entered the traps, we constructed a longitudinal dataset to reflect the strategy of primary outcome measurement in this trial. Using the time and date records, we defined variables "calendar week" and "hour of the day" and chose "Thursday, 7 am" every week from July to September to construct the longitudinal data (Figure 5). We summarised mosquito detection binary records into a count variable representing the number of mosquitoes (*Aedes* or *Culex*) entering the traps between 7 – 8 am on Thursday each week from July to September 2022. If no records were made by a given trap in this timeframe, we generated zero values to complete the data. The final datasets contained information on 12 measures of *Aedes* and *Culex* mosquito counts for five smart traps (Figure 6).


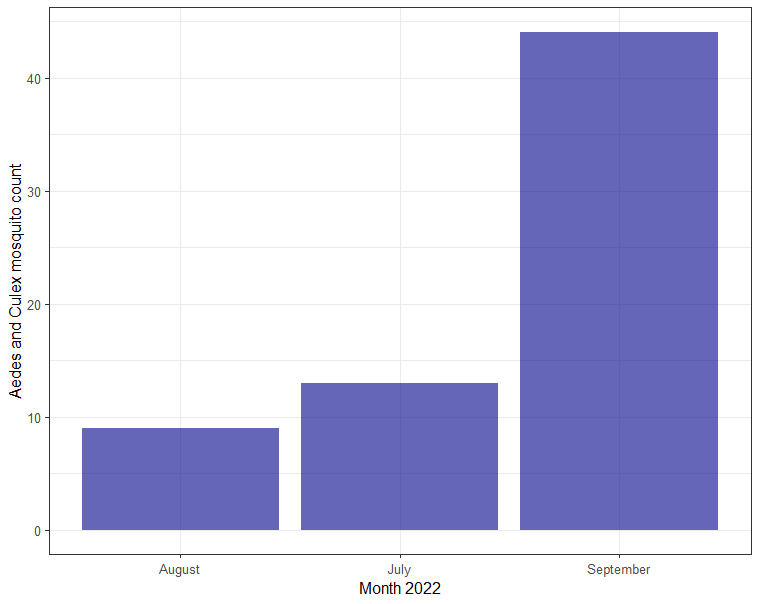

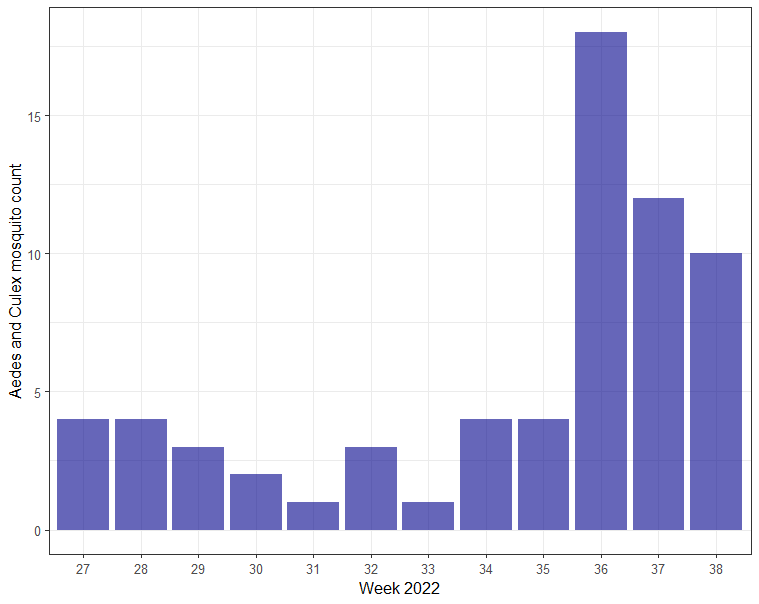


Figure 5: Aggregated counts by month (left) and week (right) pooled across all five traps.

We analysed the count data for within-subject correlation and distribution using generalised estimating equations (GEEs) and generalised linear models (GLMs), respectively.


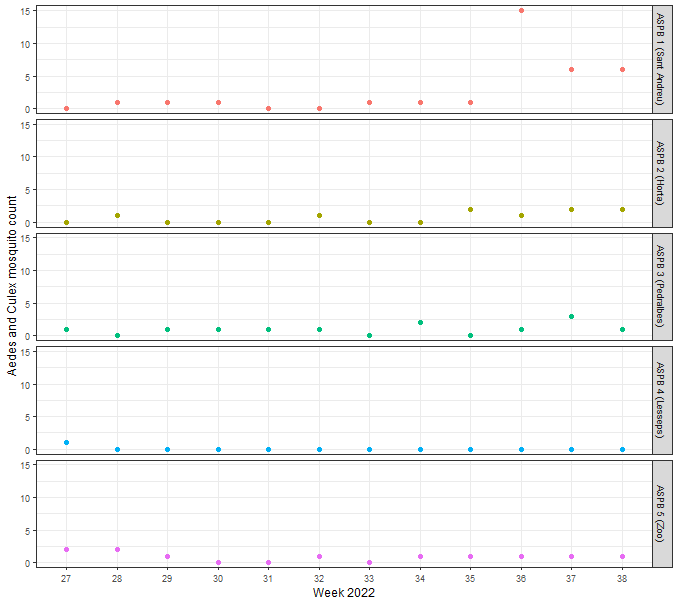


Figure 6: The counts of *Aedes* and *Culex* adult mosquitoes recorded during 2022 by the ASPB smart traps located at five sites in Barcelona.

First, we explored the within-subject correlation structure by fitting intercept-only GEEs models with the Poisson distribution family and various correlation structures: "independence", "exchangeable", "ar1", "unstructured" and "user defined". The correlation structures were analysed using QIC and QICu values for GEEs fitted to the longitudinal data. The results are given in Table 1.

*Table 1: QIC and QICu estimates for GEEs fitted to the longitudinal data using within-subject different correlation structures*

| **Correlation structure** | **QIC** | **QICu** |
| --- | --- | --- |
| Independence | 123.430 | 121.420 |
| Autoregressive 1 (AR1) | 121.149 | 121.497 |
| Exchangeable | 121.400 | 121.400 |
| Unstructured | 130.082 | 131.595 |

*Lower values suggest a better model fit

Based on the QIC/QICu values we concluded that the independence could be assumed for the within-subject correlation structure, i.e., we assumed that there is no time dependence between consecutive measurements of the mosquito counts obtained from the same mosquito trap over the course of 12 weeks. This justifies treating each observation as independent.

Thus, we analysed the count data under the assumption of independence.


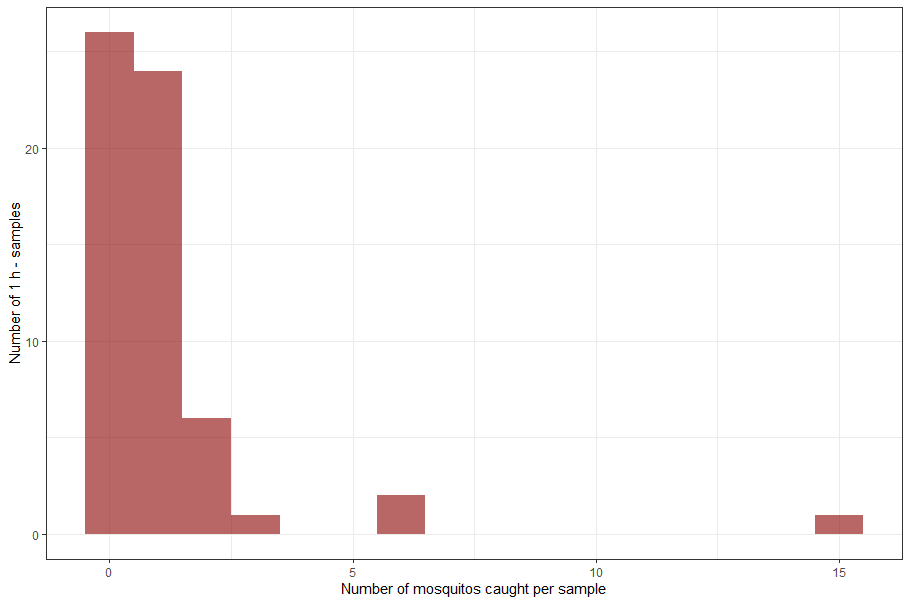


Figure 7: Distribution of mosquito count data stemming from the constructed longitudinal dataset

We fitted intercept-only GLMs with Poisson, Negative Binomial, Zero-inflated Poisson families to the weekly data from the five traps. Based on AIC estimated for the three fitted GLMs (Table 2), we concluded that the distribution of the weekly-collected count data is well approximated by the Negative Binomial distribution (Figure 7) with a mean of 1.1, variance of 2.56, and dispersion of 0.831.

*Table 2: AIC values for GLMs*

| **Model** | **AIC** |
| --- | --- |
| Poisson | 215 |
| Negative Binomial | 178 |
| Zero-inflated Poisson | 211 |

We performed the analyses in *R* using the following packages: (i) R package "geepack" (31) was used for GEE to explore the correlation structure; and (ii) R package "MASS" (32) was used for GLM with the Negative Binomial distribution.

Based on the results of the analyses of the within-subject correlation and the distribution of count outcomes, we performed the power analysis using a Negative Binomial distribution with mean and overdispersion parameters obtained from the coefficients of GLM fitted to 2022 data under the assumption of independence.

For that, we calculated power for two scenarios assuming the minimal detectable effect size of 35%, 5% significance level, and (i) sample size of 264 (22 experimental units x 12 weeks of observations due to the independence assumptions of the within-subject outcome measurements) per arm, and (ii) sample size of 369 (22 experimental units x 18 weeks of observations). The power calculation was performed in R using power_NegativeBinomial function of the *R* "PASSED" package (33). The results are given in Figure 8.


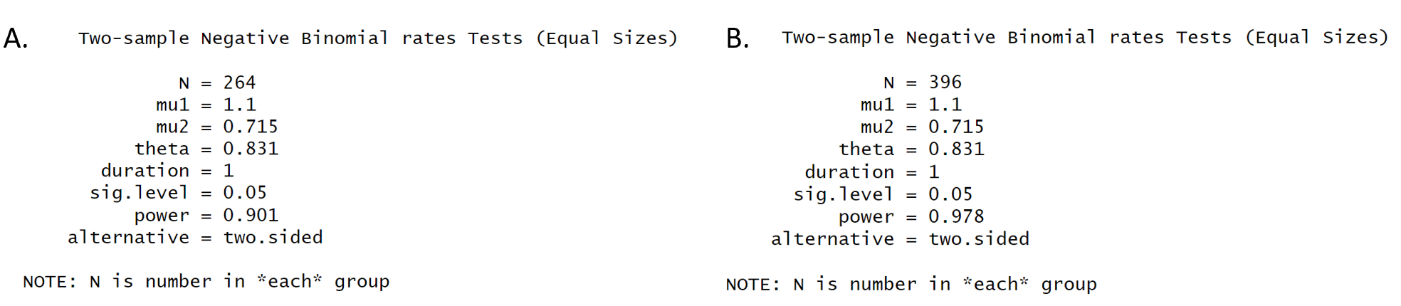


Figure 8: Results of the power calculation corresponding to (A) sample size of 264 (22 experimental units x 12 weeks of observations due to the independence assumptions of the within-subject outcome measurements) per arm, and (B) sample size of 369 (22 experimental units x 18 weeks of observations).

Based on our power analysis, we could conclude that a sample of 22 drains per arm and 12 weekly primary outcome measurements are sufficient to reach 90% power.

To note, Barcelona experienced unprecedented warmth during summer 2022 (compared to a 1971-2000 baseline), with five heat wave events, increases in the urban heat island effect, and over 40 hot nights (34,35). Summer 2023 is also set to be very warm, with record-breaking temperatures already recorded (36). Given the non-linear relationship between temperature and *Ae. albopictus* and *Cx. pipiens* activity (6), it is hard to know how our counts during 2023 will differ from those observed in 2022, and, thus, whether our statistical power will be higher or lower than estimated. So far, however, total Barcelona smart trap captures during 2023 have been higher during the year to date at the time of writing compared to 2022, suggesting higher statistical power.
